# Experimental Demonstration of Geometric Tilt-to-Length Noise Model in Test Mass Interferometer

**DOI:** 10.3390/s26134111

**Published:** 2026-06-29

**Authors:** Mengyang Zhao, Jia Shen, Shaoxin Wang, Keqi Qi, Heshan Liu, Peng Xu, Ruihong Gao, Ziren Luo

**Affiliations:** 1 School of Fundamental Physics and Mathematical Sciences, Hangzhou Institute for Advanced Study, University of Chinese Academy of Sciences (UCAS), Hangzhou 310024, China; zhaomengyang20@mails.ucas.ac.cn; 2 National Space Science Center, Chinese Academy of Sciences, Beijing 100190, China; 3 National Microgravity Laboratory, Institute of Mechanics, Chinese Academy of Sciences, Beijing 100190, China; shenjia@imech.ac.cn (J.S.); wangshaoxin@imech.ac.cn (S.W.); qikeqi@imech.ac.cn (K.Q.); liuheshan@imech.ac.cn (H.L.); xupeng@imech.ac.cn (P.X.); luoziren@imech.ac.cn (Z.L.); 4 University of Chinese Academy of Sciences, Beijing 100049, China

**Keywords:** tilt-to-length coupling, test mass interferometer, experimental verification, gravitational wave detection, differential wavefront sensing

## Abstract

Space-based gravitational wave detection missions impose extremely stringent requirements on the measurement precision of the laser interferometer, where tilt-to-length coupling noise emerges as a critical factor degrading performance. This paper focuses on geometric tilt-to-length noise in the test mass interferometer, conducting both theoretical modeling and experimental validation. First, based on the principles of geometrical optics, an analytical expression is derived for the optical path length difference variation induced by test mass angular jitter, clarifying the coupling mechanisms of the various system parameters to the tilt-to-length coupling. Numerical simulations demonstrate an excellent agreement between the theoretical model and simulation results. To further validate the theoretical model, an experimental system combining laser heterodyne interferometry and differential wavefront sensing technique is designed and constructed, with a fast steering mirror employed to simulate test mass angular jitter, enabling precise measurement of both the optical path and angular variations. By varying the lateral displacement $d_{\mathrm{lat}}$ of the fast steering mirror, the experimental data exhibit strong consistency with the theoretical prediction of the first-order tilt-to-length coupling coefficient, with a linear fitting error as low as 1.5%. Moreover, the independence of the second-order and zero-order terms relative to $d_{\mathrm{lat}}$ also aligns with the theoretical expectation. Thus, the first experimental verification of the geometric tilt-to-length coupling model is presented in this paper.

## 1. Introduction

**Figure 1:**
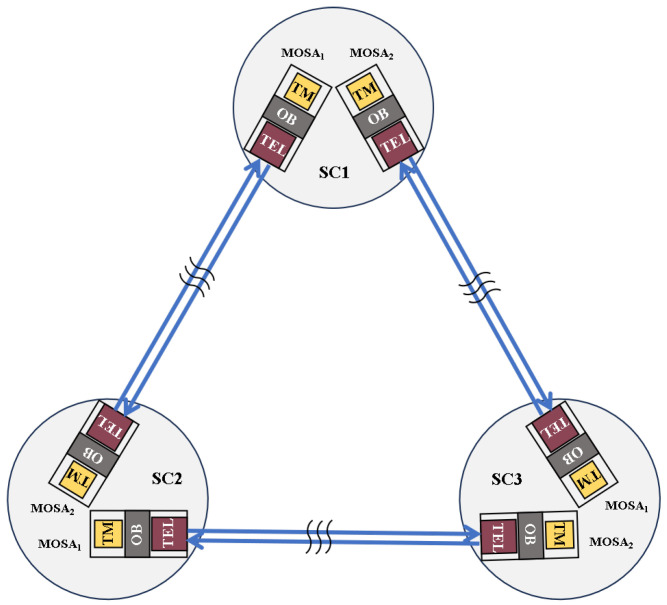
Schematic diagram of the space-based gravitational wave detection constellation links. The arrows indicate the direction of light propagation. TM: test mass, OB: optical bench, TEL: telescope.

Space-based gravitational wave detection missions aim to capture intermediate-frequency gravitational wave signals in the range of 0.1 mHz to 1 Hz through the high-precision laser interferometers in space, enabling the study of the major scientific questions such as the mergers of massive black holes, galaxy evolution, and early-universe inflation [1]. Compared with the current ground-based detectors, consisting of Advanced LIGO (aLIGO) [2], Advanced Virgo (AdVirgo) [3], and KAGRA [4], space-based detection can be carried out far from the seismic and gravity gradient noise interference, enabling observations over a broader gravitational-wave frequency band and of a wider variety of gravitational-wave sources, providing a new window for understanding the extreme astrophysical processes and the nature of gravity. Currently, the most representative space-based gravitational-wave missions include the Laser Interferometer Space Antenna (LISA) program led by the European Space Agency (ESA) [5], as well as the Taiji program [6] and Tianqin program [7] proposed by China. The core measurement principle of these missions involves detecting the tiny distance variations between two satellites through laser interferometry. Taking LISA as an example, three satellites are planned to form an equilateral triangle laser constellation with the arm lengths of millions of kilometers. When gravitational wave passes, spacetime curvature causes fluctuations in the propagation distance of light beams between two inertial reference frames in space and the fluctuations can be inferred from the phase variations in laser interference signals [5]. As shown in Figure 1, each satellite houses two moving optical sub-assemblies (MOSAs) with an included angle of approximately 60° (which varies with orbital motion). Each MOSA consists of three core payloads: a telescope, an optical bench (OB) and an inertial sensor. The test masses (TMs) placed inside the electrode cage of the inertial sensor serve as inertial references and will follow the geodesic motion when undisturbed by external forces [1,8]. To enable observations of low-frequency gravitational waves in the milli-Hz band, which are inaccessible to ground-based detectors and targeted by space-based missions [5,6], the laser interferometric system must achieve a displacement sensitivity of approximately 1 pm$/\sqrt{\mathrm{Hz}}$ over the frequency range 0.1 mHz–1 Hz.

**Figure 2:**
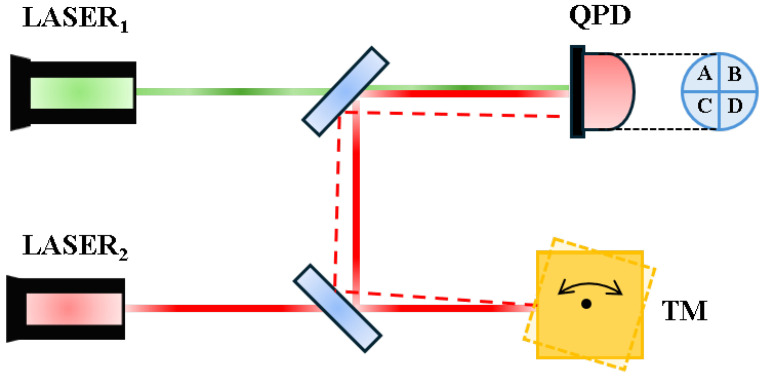
Simplified diagram of the TM interferometer. The yellow solid rectangle represents the nominal position of the TM, whereas the yellow dashed rectangle indicates its actual position. The red dashed line represents the propagation path of the beam emitted by ${\mathrm{LASER}}_{2}$ after reflection from the TM at its actual position.

The OB on each satellite is configured with three specialized laser interferometers: the science interferometer, TM interferometer and reference interferometer. The science interferometer measures the interference signal between the far-field received beam and the local reference beam, enabling the detection of the distance variations between the OBs. The TM interferometer measures the distance fluctuation of the TM relative to the local OB, and the reference interferometer is dedicated to suppressing the internal noise sources through the common-mode noise rejection techniques [9]. To meet the measurement precision requirement of 1 pm$/\sqrt{\mathrm{Hz}}$ magnitude, all the potential noise sources in the system must be suppressed to levels below this threshold. Among the identified noise contributors, the tilt-to-length (TTL) coupling noise originating from the coupling between the beam jitter and optical path length (OPL) changes has emerged as the second largest contributor to the laser interferometry noise [10]. Any source of beam jitter within the system can give rise to TTL noise. As an example, the simplified schematic of the TM interferometer is shown in Figure 2. The output beam from ${\mathrm{LASER}}_{2}$ is reflected by the TM and interferes with the beam from ${\mathrm{LASER}}_{1}$ at the quadrant photodetector (QPD). During on-orbit operation, electrostatic actuation may induce angular jitter of the TM, causing variations in the beam propagation path and consequently changes in the OPL. These OPL variations are directly transferred to the interferometric measurement, thereby reducing the accuracy of the displacement readout [11]. If left uncontrolled, the TTL noise will far exceed the required sensitivity level. Thus, research on TTL coupling models and suppression techniques is of paramount importance.

Hartig et al. have conducted a comprehensive analysis of the generation mechanisms of the TTL coupling from both geometric [12] and non-geometric perspectives [13], proposing an analytical model for TTL coupling noise in the LISA Pathfinder (LPF) mission [14]. Armano et al. [15] analyzed the LPF data before and after the realignment, identifying that TTL coupling primarily results from the relative position changes of the TMs. To validate the theoretical model, researchers have performed numerical simulations of the TTL coupling in TM interferometers. Tröbs et al. [16] investigated the sensitivity of TTL coupling to the beam parameters through simulations; Zhao et al. [17] conducted simulation analyses of TTL coupling under the combined effects of various beam and detector parameters, providing preliminary validation of the theoretical model. However, the current research on TTL noise model remains primarily focused on the analytical derivations and numerical validations, with a lack of experimental verification.

Based on the current research on noise models, the suppression methods for the TTL noise can be categorized into three main approaches. The first approach introduces a conjugate imaging system into the optical path [10,11,16,18,19,20,21] to suppress the lever arm effect of the TTL coupling. While this method effectively mitigates the primary components of the TTL noise, it cannot eliminate the geometric effect caused by the misalignment between the rotation center of TM and the beam reflection point in the TM interferometer [22], commonly denoted as piston effect. The second approach artificially introduces additional non-geometric TTL noise by deliberately creating the alignment errors of the QPD or imaging system [11,16,19,20], aiming to partially neutralize the original TTL noise. However, these alignment errors can increase the measurement errors of the differential wavefront sensing (DWS) angle and reduce the interference efficiency [23], struggling to eliminate the geometric TTL noise in the TM interferometer of practical applications. The third approach integrates the TTL noise model with the time-delay interferometry (TDI) algorithm [9,24,25,26], collecting the displacement noise data and angular data from the DWS signal, and then employing the linear fitting to determine the first-order coefficients of TTL noise for post-processing subtraction. This method can effectively eliminate the geometric TTL noise in the TM interferometer based on hardware design. However, since both the orthogonal angular directions contribute to the TTL noise in measurement, obtaining accurate first-order coefficients through fitting requires extensive in-orbit measurement data. If we can utilize the constructed noise model to estimate these coefficients, it could significantly reduce the required data volume and shorten the time needed for true value searching.

Comprehensive research demonstrates that the most effective approach for mitigating geometric TTL noise in TM interferometer is the adoption of a composite suppression strategy that combines imaging systems with TDI post-processing [9,24,26]. This approach first suppresses the dominant noise components through the optical design, and then eliminates the residual terms through post-processing algorithms. Notably, the efficiency and accuracy of TDI post-processing are directly dependent on the precision of the noise mathematical model. However, current research on TTL noise modeling primarily focuses on analytical derivations and simulation validations. The simulation validations are constrained by the simplified assumptions of computer models, resulting in the deviations from real physical systems. This disconnect between the theory and experiment hampers the engineering application of noise suppression technology, highlighting the urgent need for further experimental validation of the models. Therefore, this paper presents the first experimental verification of the geometric TTL noise model in the TM interferometer.

The paper is organized as follows: The theoretical model of the geometric TTL coupling is obtained and the accuracy of the analytical expressions is verified through the numerical simulation in Section 2. To further validate the theoretical model, an experimental system is developed. Both the design and performance of the system are introduced in Section 3. In Section 4, we present the experimental results, while the overall conclusions are shown in Section 5.

## 2. Geometric TTL Noise Model

**Figure 3:**
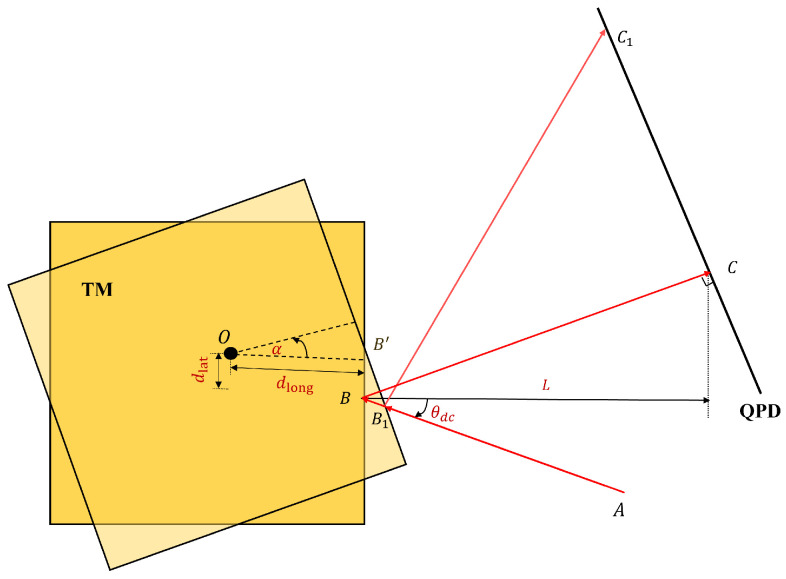
Schematic diagram of the geometric OPL variation caused by the TM angle change. Here, *O* is the jitter rotation center of the TM; *B* represents the reflection point of the measurement beam before rotation; $B^{\prime }$ is the intersection point of the TM reflecting surfaces before and after rotation; and $B_{1}$ denotes the reflection point after rotation. $d_{\mathrm{long}}$ and $d_{\mathrm{lat}}$ represent the longitudinal and lateral position deviations of the TM, respectively. The parameter α denotes the rotation angle about point *O*, while $\theta_{\mathrm{dc}}$ is the initial incidence angle deviation of the measurement beam. *L* represents the horizontal distance between the TM and the QPD when $\theta_{\mathrm{dc}}$ = 0. Points *C* and $C_{1}$ correspond to the intersection positions of the reflected beam on the QPD before and after TM rotation, respectively.

As the theoretical foundation and experimental verification object of this paper, the geometric TTL noise model in the TM interferometer will be established in this section. To better understand and more clearly describe the TTL coupling process, Figure 2 is further simplified by retaining only the TM and detector, while shortening the optical path of the fixed reference beam, as shown in Figure 3. Although the attitude and translational control of the TM are inherently complex, in the analysis of TTL coupling, the TM can be reasonably approximated as a rigid body that is free to undergo arbitrary rotations about any point as well as translations. TM is a metal cube whose jitter rotation center *O* typically does not coincide with the measurement beam reflection point *B* during in-orbit motion, resulting in the longitudinal and lateral position deviations $d_{\mathrm{long}}$ and $d_{\mathrm{lat}}$. The measurement beam is not perfectly perpendicularly incident on the TM surface, exhibiting an initial incidence angle deviation $\theta_{\mathrm{dc}}$, while *L* represents the horizontal distance between the TM and QPD when there is no $\theta_{\mathrm{dc}}$.

At the initial moment, the measurement beam follows the path A→B→C; after the TM rotates by an angle α around point *O*, *O* is the jitter rotation center of TM which is not necessarily the center of TM, the path of measurement beam changes to $A\to B_{1}\to C_{1}$, and the reflecting surfaces of the TM before and after rotation intersect at point $B^{\prime }$. Thus, a variation in the OPL that is dependent on the angular fluctuation α is generated and the TTL coupling is induced. Next, we will analyze the theoretical model to derive an analytical expression and perform numerical simulation to preliminarily verify the results.

Based on the geometric relationships, the distance $BB^{\prime }$ can be readily derived as:

(1) $$BB^{\prime }=d_{\mathrm{lat}}+d_{\mathrm{long}}\tan\frac{\alpha }{2}.$$

Consequently, the distance $BB_{1}$ between the reflection points before and after the TM rotation can be calculated as:

(2) $$BB_{1}=BB^{\prime }\frac{\sin\alpha }{\cos(\theta_{\mathrm{dc}}+\alpha )}=\frac{\left(d_{\mathrm{lat}}+d_{\mathrm{long}}\tan\frac{\alpha }{2}\right)\sin\alpha }{\cos(\theta_{\mathrm{dc}}+\alpha )}.$$

According to the beam reflection principle:

(3) $$BC=\frac{L}{\cos\theta_{\mathrm{dc}}},$$

Then, through the geometric relationships, the vertical distance from the rotated reflection point $B_{1}$ to the QPD can be calculated in the following:

(4) $$B_{1}C_{1}=\frac{L}{\cos\theta_{\mathrm{dc}}\cos2\alpha }-\frac{\left(d_{\mathrm{lat}}+d_{\mathrm{long}}\tan\frac{\alpha }{2}\right)\sin\alpha \cos2\theta_{\mathrm{dc}}}{\cos(\theta_{\mathrm{dc}}+\alpha )\cos2\alpha }.$$

In summary, the optical path length difference (OPD) before and after the TM jitter can be obtained as:

(5) $$\begin{array}{cc} \mathrm{OPD} & =AB_{1}+B_{1}C_{1}-\left(AB+BC\right)\\ \; & =\frac{2L\sin^{2}\alpha }{\cos\theta_{\mathrm{dc}}\cos2\alpha }-\left(d_{\mathrm{lat}}+d_{\mathrm{long}}\tan\frac{\alpha }{2}\right)\frac{2\cos(\theta_{\mathrm{dc}}-\alpha )\sin\alpha }{\cos2\alpha }. \end{array}$$

This derivation accounts for the actual OPL changes before and after the jitter, thus the result incorporates both the lever arm and piston effects of the TTL noise.

In the space-based gravitational wave detection missions, the maximum in-orbit angular tilt α of the TM is only on the order of hundreds of microradians. Moreover, $\theta_{dc}$ is also assumed to be sufficiently small. Therefore, the Taylor series to second-order near zero is performed, yielding the following approximate relationship:

(6) $$\mathrm{OPD}\approx -d_{\mathrm{lat}}\left(2-\theta_{\mathrm{dc}}^{2}\right)\cdot \alpha +\left(2L-d_{\mathrm{long}}-2d_{\mathrm{lat}}\theta_{\mathrm{dc}}\right)\cdot \alpha^{2}.$$

Through taking the derivative of both sides of the equation above, the analytical expression for TTL coupling noise is determined as:

(7) $$d\mathrm{OPD}\approx -d_{1\mathrm{at}}\left(2-\theta_{\mathrm{dc}}^{2}\right)\cdot d\alpha +2\left(2L-d_{\mathrm{long}}-2d_{1\mathrm{at}}\theta_{\mathrm{dc}}\right)\cdot \alpha d\alpha ,$$

where dα represents the angular jitter magnitude, and dOPD is the resulting displacement noise due to the jitter.

Under the electrostatic control, the angular jitter of the TM dα is on the order of 100 nrad$/\sqrt{\mathrm{Hz}}$. The rotation center of the TM is typically near its center of mass, so $d_{\mathrm{long}}$ is primarily determined by the TM dimensions, on the order of 0.1 m. *L* is some factor that would be distance to the QPD without a lens but that even with imaging optics in place should have dimensions less than total (non-imaged) optical pathlength between TM and QPD; i.e., we use *L* = 1 m later as an upper bound for the approximation. The parameters $d_{\mathrm{lat}}$ and $\theta_{\mathrm{dc}}$ depend on the alignment errors between the TM and the optical bench, which can achieve 10 μm/10 μrad magnitude with current alignment capabilities. Considering that the noise budget of space-borne gravitational wave detections for TTL coupling noise is 1 pm$/\sqrt{\mathrm{Hz}}$, the contribution factors smaller than 0.1 pm$/\sqrt{\mathrm{Hz}}$ are excluded through estimating the actual magnitudes of each system parameter; thus, the above equation can be further simplified as:

(8) $$d\mathrm{OPD}\approx -2d_{1\mathrm{at}}\cdot d\alpha +2\left(2L-d_{\mathrm{long}}\right)\cdot \alpha d\alpha .$$

The TTL coupling coefficient $k_{\Delta s-\alpha }$, defined as the ratio of dOPD to dα, serves as a critical metric for evaluating the sensitivity of OPD to angular fluctuations and is the core parameter in assessing TTL noise magnitude. From the above equation, the expression for $k_{\Delta s-\alpha }$ and the simplified OPD can be derived as:

(9) $$k_{\Delta s-\alpha }=\frac{d\mathrm{OPD}}{d\alpha }\approx -2d_{\mathrm{lat}}+2\left(2L-d_{\mathrm{long}}\right)\alpha ,$$

(10) $$\mathrm{OPD}\approx -2d_{\mathrm{lat}}\alpha +\left(2L-d_{\mathrm{long}}\right)\alpha^{2}.$$

(11) $$\mathrm{OPD}\approx k_{1}\alpha +k_{2}\alpha^{2},$$

where $k_{1}\;=\;-2d_{\mathrm{lat}}$, $k_{2}\;=\;2L-d_{\mathrm{long}}$. It is evident that $d_{\mathrm{lat}}$ contributes to the first-order TTL coupling, while the combination of L and $d_{\mathrm{long}}$ introduces the second-order TTL coupling, with the influence of $\theta_{\mathrm{dc}}$ being negligible. Considering the practical space missions, we adopt the following parameter values: lateral displacement $d_{\mathrm{lat}}$ = 50 μm, longitudinal displacement $d_{\mathrm{long}}$ = 23 mm which is equal to half of the side length of the TM [27], angular deflection α∈ [−200 μrad, 200 μrad] and horizontal distance *L* = 1 m. Under this condition, the maximum values of the OPD and the TTL coupling coefficient $k_{\Delta s-\alpha }$ are approximately 100 nm and 910 μm/rad, respectively, far exceeding the requirements of both the Taiji program (5 μm/rad@ ± 200 μrad [22]) and the LISA program (25 μm/rad@ ± 300 μrad [16]). Although the second-order term may contribute more significantly to the TTL noise, it can, in principle, be substantially suppressed or even eliminated through the implementation of imaging systems and optimization of the optical installation and alignment. The effectiveness of suppressing the second-order coupling has been demonstrated and experimentally verified in previous studies [10,16,18,19]. However, even after the second-order contribution has been mitigated, the residual first-order term still reaches 100 μm/rad which remains significantly beyond the requirement. More importantly, unlike the second-order term, there is currently no direct optical design approach capable of effectively suppressing the first-order coupling. Consequently, post-processing algorithms are required to further reduce the associated TTL noise. The primary objective of this work is therefore to experimentally validate the first-order TTL noise model, thereby providing a foundation for the development and implementation of subsequent TTL noise subtraction and suppression techniques.

**Figure 4:**
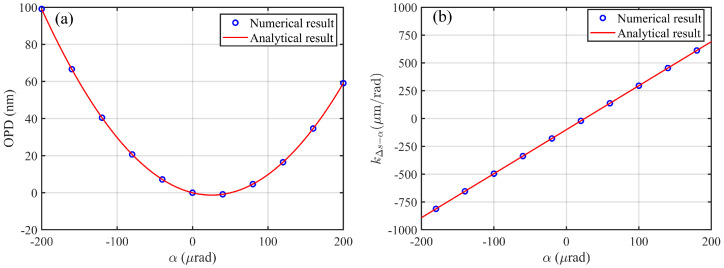
(**a**) relationship between the OPD and the TM deflection angle α; (**b**) relationship between $k_{\Delta s-\alpha }$ and the TM deflection angle α. The red curves represent the analytical results, while the blue circles are numerical results.

We first conduct a preliminary verification of the analytical expression through numerical simulation. For the aforementioned system parameters, a detailed optical model of the geometric OPL variation caused by the TM angle change is established using the ASAP NextGen V1 SP2 optical simulation software [28]. Geometric ray tracing is employed to simulate the propagation of the laser beams. TM jitter is modeled as angular perturbations of the test mass relative to its nominal orientation. The OPLs corresponding to the nominal and perturbed configurations are calculated separately, and the OPD and TTL coupling results are obtained from their differences. Subsequently, we utilize MATLAB R2018a [29] for numerical computation and analysis, yielding the corresponding OPD and TTL coupling results as shown in Figure 4. The results demonstrate that the theoretical predictions closely match the simulation outcomes under these system parameters, with a maximum residual error of only 6 fm, preliminarily confirming the correctness of the theoretical model. Next, we will proceed with experimental validation by constructing an experimental system.

## 3. Experimental System

### 3.1. System Setup

**Figure 5:**
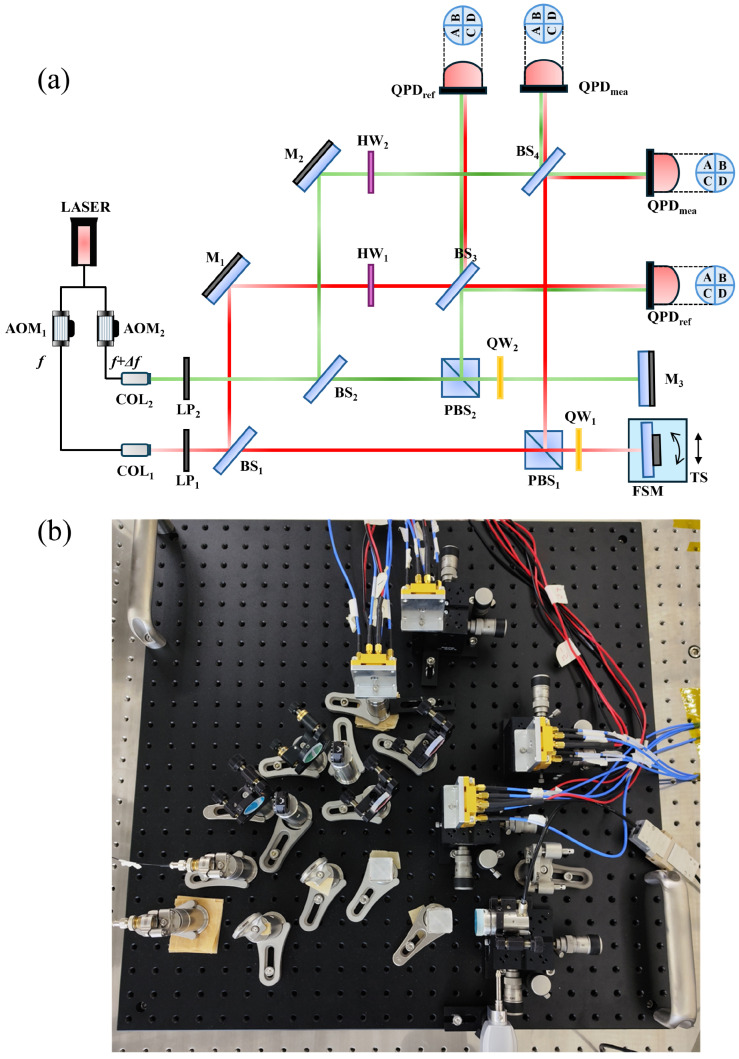
(**a**) Diagram of the experimental system for calibrating the TTL noise model. BS: Beam Splitter; PBS: Polarizing Beam Splitter; AOM: Acousto-Optic Modulator; COL: Fiber Collimator; LP: Linear Polarizer; M: Mirror; FSM: Fast Steering Mirror; TS: Translation Stage; QW: Quarter Waveplate; HW: Half Waveplate; QPD: Quadrant Photodetector; A-D denote the four quadrants of the QPD. (**b**) Photograph of the experimental setup.

To validate the geometric TTL noise model derived in Section 2, an experimental system based on laser heterodyne interference is designed, as shown in Figure 5.

The laser beam is split by a 1 × 2 fiber coupler and connected to two Acousto-Optic Modulators (AOMs), which introduce a frequency difference Δf (Δf=1.6MHz in the experiment) after modulation. Fiber collimators are connected to the AOM outputs to direct the beams to the optical platform, where the red beam represents the measurement beam and the green beam represents the reference beam. The measurement beam passes through ${\mathrm{BS}}_{1}$ and ${\mathrm{PBS}}_{1}$ in turn, and is reflected by a fast steering mirror (FSM) which simulates TM motion. It then passes through ${\mathrm{QW}}_{1}$ twice, changing its polarization from p to s, and is consequently reflected when passing through ${\mathrm{PBS}}_{1}$ again. The reference beam follows a similar path, except that the FSM is replaced by a mirror $M_{3}$. The two beams are interfered at ${\mathrm{BS}}_{3}$ and ${\mathrm{BS}}_{4}$, and hit toward ${\mathrm{QPD}}_{\mathrm{ref}}$ and ${\mathrm{QPD}}_{\mathrm{mea}}$, respectively, forming a reference interferometer and a measurement interferometer. (The laser wavelength is 1064 nm. The beam waist diameter is 1.5 mm, and after propagation, the beam diameter at the detector surface is approximately 1.61 mm. The active-area diameter of the QPD used in the experiment is 1.2 mm.) The measurement interferometer is used to detect the TTL noise caused by the angular changes of the FSM, while the reference interferometer is used to subtract the common-mode environmental noise from the measurement interferometer. Both interferometers are designed with equal arms to minimize the impact of the laser frequency noise.

The QPDs convert optical signal into electrical signal. Then, a phase meter is used to analyze the phase of the signal. In this experiment, a self-developed phase meter based on digital phase-locked loop (DPLL) technique is used, with a phase measurement precision of 2π μ$\mathrm{rad}/\sqrt{\mathrm{Hz}}$ @ (1 mHz–1 Hz), meeting the experimental requirement. According to the high-precision phase values ($\varphi_{A}$, $\varphi_{B}$, $\varphi_{C}$, $\varphi_{D}$) of the four-quadrant heterodyne interference signals measured through the phase meter and the wavenumber *k*, the longitudinal pathlength signal (LPS) can be obtained as follows:

(12) $$\mathrm{LPS}=\frac{\varphi_{A}+\varphi_{B}+\varphi_{C}+\varphi_{D}}{4k}.$$

The LPS signal will change when the FSM rotates, and the primary contributor is the OPD induced by the TTL noise. In the experiment, the FSM is driven by a Physik Instrumente (PI), Karlsruhe, Germany, S-330.2SL piezoelectric tilt stage, which enables yaw and pitch angle adjustments with a deflection range of 2 mrad and an angular resolution of 50 nrad, meeting the requirement for simulating the TM angular jitter in this experiment. To accurately measure the actual angular variations at the QPD after adjusting the FSM, the DWS technology is employed, which has been widely applied in the laser interferometers for space-based gravitational wave detection missions. When the angle between the two interfering beams is not large (typically considered within ±400 μrad), the DWS signal exhibits an approximately linear relationship with the corresponding beam angle [30]; thus, the high-precision phase measurement results can be converted into high-precision angular measurements. Taking the yaw direction as an example, the expression is as follows:

(13) $${\mathrm{DWS}}_{rl}=\frac{\varphi_{B}+\varphi_{D}-\varphi_{A}-\varphi_{C}}{2}\approx k_{rl}\cdot \alpha ,$$

where α represents the beam angle, and $k_{rl}$ is the DWS phase-to-angle conversion coefficient.

To verify Equation (10), it is necessary to experimentally sweep the first-order coefficient $d_{\mathrm{lat}}$; thus, we mounted the piezo-tilt stage on a MicroBlock translation stage from Thorlabs company (Newton, NJ, USA). This translation stage enables three-axis (X,Y,Z) adjustment with a maximum travel range of 4 mm and is equipped with a precision differential adjuster, where each revolution of the fine adjustment provides a resolution of 50 μm. To accurately calibrate the variation of $d_{\mathrm{lat}}$, a Mitutoyo Corporation, Kawasaki, Japan, Mitutoyo 543-491B digital dial indicator with a measurement resolution of 1 μm was used in the experiment.

### 3.2. System Performance

**Figure 6:**
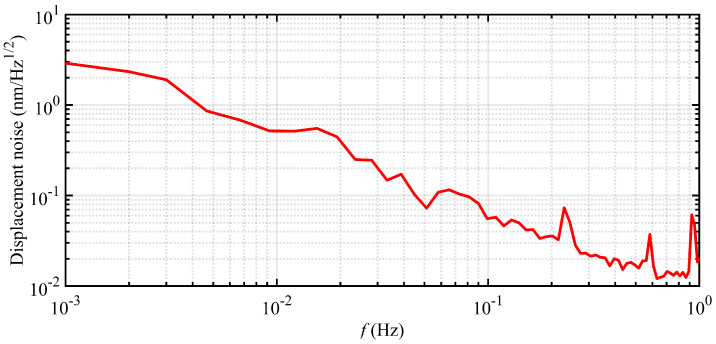
The amplitude spectral density of the displacement noise for the TM interferometer in the static state.

Based on the above design, we constructed the experimental system and tested the performance. We first evaluated the background noise level of the laser interferometric measurement system in an air-tight environment, with the FSM held at its initial position for the static data acquisition. By subtracting the reference interferometer results from the measurement interferometer results to eliminate the common-mode noise, we obtained the results shown in Figure 6, achieving a noise floor of 60 pm$/\sqrt{\mathrm{Hz}}$@0.1 Hz. In the TTL noise model calibration experiment, we measured the LPS signal variation caused by the angular changes, thus focusing on the time-domain measurement results. The data in Figure 6 corresponds to a displacement noise RMS (Root Mean Square) value of approximately 0.87 nm over a 200 s period. According to a rough estimation using Equation (10) and the designed system parameters, even in the absence of $d_{\mathrm{lat}}$ and $d_{\mathrm{long}}$, a 200 μrad angular offset would induce an OPD on the order of 10 nm, which is significantly larger than the experimentally measured displacement noise RMS value. Therefore, the displacement measurement performance of the experimental system meets the requirement for noise model calibration.

**Figure 7:**
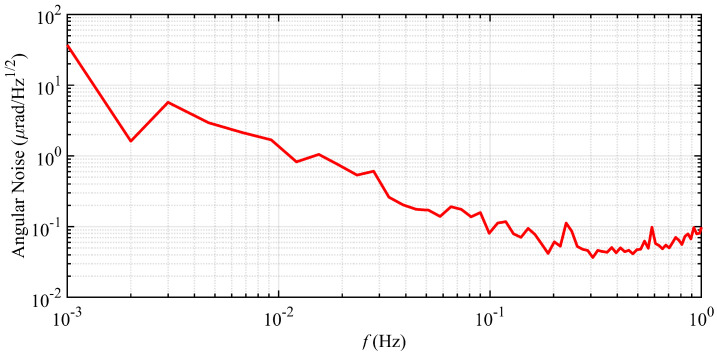
The amplitude spectral density of the yaw-direction angle noise for the TM interferometer measured through the DWS signal in the static state.

We then evaluated the angular measurement capability of the experimental system. First, we controlled the piezo-tilt stage to achieve the specific angular rotations of the FSM and calculated the corresponding DWS signals at different FSM jitter angles through the phase meter, and subsequently fitted the DWS coefficient. Taking the yaw direction as an example, we experimentally calibrated the DWS coefficient to be $k_{rl}$ = 2606 rad/rad through this method. Given the phase measurement capability of 2π μ$\mathrm{rad}/\sqrt{\mathrm{Hz}}$, the theoretical angular measurement resolution can reach 2.4 nrad$/\sqrt{\mathrm{Hz}}$. However, due to the environmental factors and fiber collimator jitter, the actual angular background noise for the yaw direction of the TM interferometer is shown in Figure 7. Although this is somewhat worse than the theoretical value, it still achieves 90 nrad$/\sqrt{\mathrm{Hz}}$@0.1 Hz, which fully meets the angular measurement requirement for the noise calibration experiment.

### 3.3. Process Design

After completing the experimental system setup and performance calibration, we proceeded to validate the noise model. The test results in the yaw direction are primarily presented in this paper, as the pitch direction yields the consistent conclusions. The detailed experimental procedure is as follows:

- Control the FSM to perform a sinusoidal scan, while the ${\mathrm{QPD}}_{\mathrm{ref}}$ and ${\mathrm{QPD}}_{\mathrm{mea}}$ continuously measure the interference signals. The phase meter separately resolves the phase values of the four quadrants for both detectors;
- Calculate the LPS signals (${\mathrm{LPS}}_{\mathrm{ref}}$ and ${\mathrm{LPS}}_{\mathrm{mea}}$) for the reference and measurement interferometers according to Equation (12). Subtract the two signals to obtain the displacement change after the common-mode noise suppression, ${\mathrm{LPS}}_{0}={\mathrm{LPS}}_{\mathrm{mea}}-{\mathrm{LPS}}_{\mathrm{ref}}$;
- Calculate the DWS signal for the measurement interferometer according to Equation (13). Use the calibrated phase-to-angle conversion coefficient $k_{rl}$ to determine the angle at the QPD, then divide the result by 2 to obtain the actual angle $\alpha_{rl}$ of the FSM;
- Plot the $\alpha_{rl}-{\mathrm{LPS}}_{0}$ relationship curve and perform quadratic fitting on the data to obtain the fitting coefficients for each order;
- Horizontally move the translation stage to vary the $d_{\mathrm{lat}}$ value of the FSM, and use the digital indicator to measure the position change;
- Repeat steps a–e to measure the changes in the fitting coefficients after introducing different $d_{\mathrm{lat}}$ variations.

## 4. Results and Discussion

**Figure 8:**
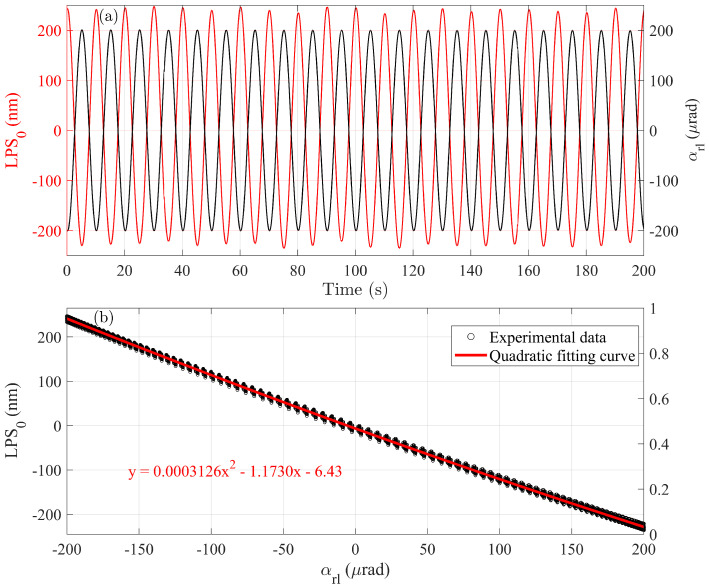
(**a**). Measurement results of ${\mathrm{LPS}}_{0}$ signal variation and $\alpha_{rl}$ variation caused by the FSM scanning; (**b**) relationship curve between $\alpha_{rl}$ and ${\mathrm{LPS}}_{0}$ at the initial position, where the black circles represent the experimental measurement data and the red curve represents the quadratic fitting curve.

Following the experimental procedure outlined in Section 3.3, we first tested the FSM in its initial position by varying the angle and measuring the resulting changes in the LPS signal. According to Equation (10), the relationship between the FSM rotation angle and the LPS signal is theoretically quadratic; thus, we performed quadratic fitting on the experimental data to obtain the fitting curve and the coefficients of each order. Considering that the requirement for the TTL coupling noise of the TM interferometer in the Taiji program is ±5 μm/rad@ ± 200 μrad, we set the sine sweep range of the FSM to (−200 μrad, +200 μrad) in the experiment, with a sweep frequency of 0.1 Hz. After continuously acquiring the interference signals for 200 s, the calculated ${\mathrm{LPS}}_{0}$ and $\alpha_{rl}$ values through Equations (12) and (13) are shown in Figure 8a, and the corresponding $\alpha_{rl}-{\mathrm{LPS}}_{0}$ relationship curve is presented in Figure 8b.

The experimental test results exhibit a certain degree of broadening along the vertical axis, which can be attributed to two main factors: environmental noise and the hysteresis effect of the piezoelectric ceramic motion. By performing the second-order fitting on data from multiple cycles, the data averaging effect was achieved which significantly reduced the impact of these factors on the calibration. The red curve represents the obtained quadratic fitting curve, with an R-square value of 0.9989, indicating excellent fitting performance.

**Figure 9:**
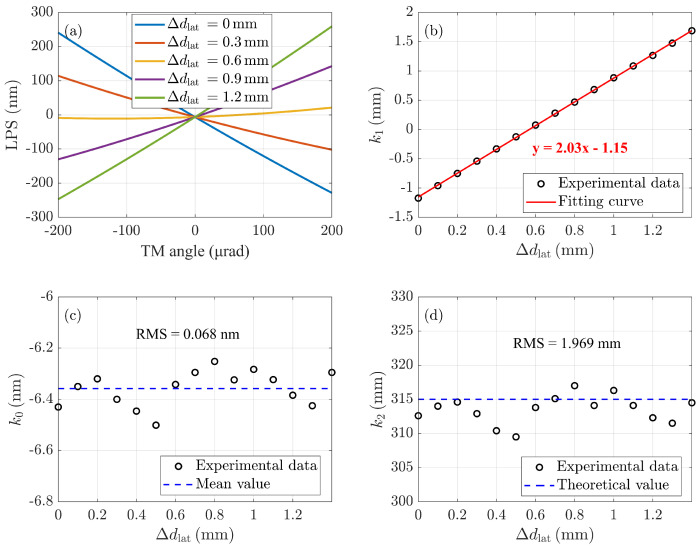
(**a**). Quadratic fitting curves of $\alpha_{rl}-{\mathrm{LPS}}_{0}$ at different $d_{\mathrm{lat}}$ positions; (**b**) first-order fitting coefficient values at different $d_{\mathrm{lat}}$ positions; (**c**) zero-order fitting coefficient values at different $d_{\mathrm{lat}}$ positions; (**d**) second-order fitting coefficient values at different $d_{\mathrm{lat}}$ positions.

We then horizontally moved the translation stage and monitored the displacement change through a digital dial indicator, varying the $d_{\mathrm{lat}}$ value of FSM in the increments of 100 μm. At each $d_{\mathrm{lat}}$ position, we repeated the aforementioned measurement process to obtain the corresponding quadratic fitting curves. A total of 15 different positions were tested in the experiment, with partial results shown in Figure 9a. Subsequently, we performed polynomial fitting of the experimental data with different orders. The results are presented in Figure 9b,c. In contrast to Equation (10), a constant offset was observed in the experimental data, which we define as the Zero-order fitting term.

Since we could not determine the initial $d_{\mathrm{lat}}$ value, we considered the displacement amount $\Delta d_{\mathrm{lat}}$ relative to the initial position $d_{\mathrm{lat},0}$ in the experiment. According to Equation (10), the zero-order coefficient $k_{0}$ and the second-order coefficient $k_{2}$ are theoretically independent of $d_{\mathrm{lat}}$, while the first-order coefficient is $k_{1}=-2d_{\mathrm{lat}}$. Considering that the first-order fitting coefficient at the initial position was negative and relatively large according to Figure 8b, we moved in the opposite direction when traversing $d_{\mathrm{lat}}$ values for better interference effects, that is $d_{\mathrm{lat}}=-\Delta d_{\mathrm{lat}}+d_{\mathrm{lat},0}$; thus, we obtained:

(14) $$k_{1}=2\Delta d_{\mathrm{lat}}-2d_{\mathrm{lat},0}.$$

The first-order coefficient and $\Delta d_{\mathrm{lat}}$ are theoretically expected to exhibit a linear relationship. Based on the experimental test results, we plotted the relationships between $\Delta d_{\mathrm{lat}}$ and the first-order, zero-order, and second-order fitting coefficients at different positions, as shown in Figure 9b, c and d, respectively.

In Figure 9b, the linear fitting of the experimental data yields $k_{1}=2.03\Delta d_{\mathrm{lat}}-1.15$, with the curve R-square value approximately equal to 1, indicating an excellent linear relationship between $k_{1}$ and $\Delta d_{\mathrm{lat}}$. The experimental results show a greatly consistent form with the theoretical predictions, with the fitting error for the first-order term being only 1.5%, which fully validates the correctness of the theoretical expression.

Based on the content of Figure 9c, the experimentally measured zero-order coefficient $k_{0}$ remains constant at approximately −6.3 nm and does not vary with changes in $d_{\mathrm{lat}}$. Theoretically, the value of $k_{0}$ should remain unchanged at 0. This discrepancy arises from the noise introduced by the initial optical path alignment errors in the experimental system, with the predominant contributing factor being the existence of an initial angle between the two interfering beams.

Based on the data from Figure 9d, the experimentally measured second-order coefficient $k_{2}$ remains constant at approximately 313 mm and also does not vary with changes in $d_{\mathrm{lat}}$, exhibiting a fluctuation RMS value of 1.969 mm. Measurements of the experimental system reveal that the optical transmission distance L between the FSM reflection point and the detector is approximately 160 mm. Given that the FSM rotation center is positioned near the front surface of the piezoelectric ceramic and the mirror thickness is 5 mm, the distance $d_{\mathrm{long}}$ between the FSM rotation center and the reflection point is approximately 5 mm. Theoretically, $k_{2}=2L-d_{\mathrm{long}}$, which calculates to a nominal value of 315 mm. The close agreement between the theoretical calculation and experimental calibration results further validates the correctness of the analytical expression. The discrepancy between the experimental and theoretical values for the second-order term arises from two sources: the background noise of the measurement system and the estimation error of the actual rotation center position of the FSM.

In summary, it is evident that the experimental findings align well with the theoretical predictions, providing strong validation for the correctness of the geometric TTL noise model in TM interferometer.

## 5. Conclusions

This paper conducts the theoretical modeling and experimental verification of the geometric TTL noise in TM interferometer for space-based gravitational wave detection missions, aiming to address the lack of experimental support in current TTL noise research. Firstly, based on geometric optics principles, the analytical expression for the OPD variation caused by TM angular jitter has been derived, clarifying that the coupling between geometric TTL noise and lateral displacement $d_{\mathrm{lat}}$ primarily manifests as a first-order term of the angle, while the coupling with longitudinal $d_{\mathrm{long}}$ and vertical distance *L* appears as second-order terms. Then the preliminary verification through numerical simulation has been performed, where the theoretical model shows excellent agreement with the simulation results, with a maximum residual error of only 6 fm. Subsequently, the experimental verification of the theoretical results was conducted. According to the theoretical predictions, the dominant first-order noise induced by $d_{\mathrm{lat}}$ cannot be eliminated by conventional methods and thus serves as the primary focus of our experimental validation. We developed an experimental system based on the laser heterodyne interferometry and DWS technology, precisely measuring the OPL and angular variations through using the FSM to simulate TM angular jitter. The experimental system achieved measurement precisions of 60 pm$/\sqrt{\mathrm{Hz}}$@0.1 Hz for displacement and 90 nrad$/\sqrt{\mathrm{Hz}}$@0.1 Hz for angle, meeting the requirements for model verification. The experimental results demonstrate that the linear relationship between the first-order TTL noise coefficient $k_{1}$ and the lateral displacement $d_{\mathrm{lat}}$, matches the theoretical predictions well, with only 1.5% error. The independence of the second-order and zero-order terms from $d_{\mathrm{lat}}$ conforms to the theoretical expectations as well. These findings fully validate the correctness of the theoretical model, providing reliable theoretical basis and experimental support for suppressing TTL noise in space-based gravitational wave detection missions, offering important guidance for optimizing optical design and post-processing algorithms.

## Data Availability

Data will be made available on request.

## References

[1] Armano M., Audley H., Auger G., Baird J.T., Bassan M., Binetruy P., Born M., Bortoluzzi D., Brandt N., Caleno M. (2016). Sub-femto-g free fall for space-based gravitational wave observatories: LISA pathfinder results. Phys. Rev. Lett..

[2] Collaboration T.L.S., Aasi J., Abbott B.P., Abbott R., Abbott T., Abernathy M.R., Ackley K., Adams C., Adams T., Addesso P. (2015). Advanced LIGO. Class. Quantum Gravity.

[3] Acernese F., Agathos M., Agatsuma K., Aisa D., Allemandou N., Allocca A., Amarni J., Astone P., Balestri G., Ballardin G. (2014). Advanced Virgo: A second-generation interferometric gravitational wave detector. Class. Quantum Gravity.

[4] KAGRA Collaboration (2019). KAGRA: 2.5 generation interferometric gravitational wave detector. Nat. Astron..

[5] Amaro-Seoane P., Audley H., Babak S., Baker J., Barausse E., Bender P., Berti E., Binetruy P., Born M., Bortoluzzi D. (2017). Laser Interferometer Space Antenna. arXiv.

[6] Luo Z., Guo Z., Jin G., Wu Y., Hu W. (2020). A brief analysis to Taiji: Science and technology. Results Phys..

[7] Luo J., Chen L., Duan H., Gong Y., Hu S., Ji J., Liu Q., Mei J., Milyukov V., Sazhin M. (2016). TianQin: A space-borne gravitational wave detector. Class. Quantum Gravity.

[8] Hartig M.S. (2022). Tilt-to-Length Coupling in LISA Pathfinder: Model, Data Analysis and Take-Away Messages for LISA. Ph.D. Thesis.

[9] Wanner G., Shah S., Staab M., Wegener H., Paczkowski S. (2024). In-depth modeling of tilt-to-length coupling in LISA’s interferometers and TDI Michelson observables. Phys. Rev. D.

[10] Chwalla M., Danzmann K., Barranco G.F., Fitzsimons E., Gerberding O., Heinzel G., Killow C.J., Lieser M., Perreur-Lloyd M., Robertson D.I. (2016). Design and construction of an optical test bed for LISA imaging systems and tilt-to-length coupling. Class. Quantum Gravity.

[11] Lee Y. (2021). Development of an Advanced Tilt Actuator for Tilt-to-Length Coupling Investigations. Ph.D. Thesis.

[12] Hartig M.S., Schuster S., Wanner G. (2022). Geometric tilt-to-length coupling in precision interferometry: Mechanisms and analytical descriptions. J. Opt..

[13] Hartig M.S., Schuster S., Heinzel G., Wanner G. (2023). Non-geometric tilt-to-length coupling in precision interferometry: Mechanisms and analytical descriptions. J. Opt..

[14] Hartig M.S., Wanner G. (2023). Tilt-to-length coupling in LISA Pathfinder: Analytical modeling. Phys. Rev. D.

[15] Armano M., Audley H., Baird J., Binetruy P., Born M., Bortoluzzi D., Castelli E., Cavalleri A., Cesarini A., Cruise A. (2023). Tilt-to-length coupling in LISA Pathfinder: A data analysis. Phys. Rev. D.

[16] Tröbs M., Schuster S., Lieser M., Zwetz M., Chwalla M., Danzmann K., Barránco G.F., Fitzsimons E., Gerberding O., Heinzel G. (2018). Reducing tilt-to-length coupling for the LISA test mass interferometer. Class. Quantum Gravity.

[17] Zhao M., Shen J., Peng X., Ma X., Yang Z., Liu H., Meng X., Zhang J. (2024). Analysis of tilt-to-length coupling noise: Exploring the influence of multiple factors in test mass interferometers. Chin. Opt..

[18] Schuster S., Tröbs M., Wanner G., Heinzel G. (2016). Experimental demonstration of reduced tilt-to-length coupling by a two-lens imaging system. Opt. Express.

[19] Chwalla M., Danzmann K., Álvarez M.D., Delgado J.E., Fernández Barranco G., Fitzsimons E., Gerberding O., Heinzel G., Killow C., Lieser M. (2020). Optical suppression of tilt-to-length coupling in the LISA long-arm interferometer. Phys. Rev. Appl..

[20] Mu H., Lin X., Xu X., Tan Y., Wei H., Li Y. (2025). Suppression of tilt-to-length coupling in transponder-type interferometer with imaging system and deliberate tilt of photodetectors. Opt. Lasers Eng..

[21] Lin X., Yan H., Miao H.X., Qiu P., Liang Y.R., Yeh H.C., Zhou Z.B. (2024). Compact advanced pure tilt actuator for testing tilt-to-length coupling in space-based gravitational wave detection. Opt. Express.

[22] Shen J., Wang S., Qi K., Zhao M., Liu H., Yang R., Li P., Tao W., Luo Z., Gao R. (2024). The Suppression Effect of an Imaging System on the Geometric Tilt-to-Length Coupling in a Test Mass Interferometer. Photonics.

[23] Gao R., Wang Y., Cui Z., Liu H., Jia J., Luo Z., Jin G. (2023). Zero-offset analysis on differential wavefront sensing technique in gravitational wave detection missions. Microgravity Sci. Technol..

[24] Paczkowski S., Giusteri R., Hewitson M., Karnesis N., Fitzsimons E., Wanner G., Heinzel G. (2022). Postprocessing subtraction of tilt-to-length noise in LISA. Phys. Rev. D.

[25] Houba N., Delchambre S., Ziegler T., Fichter W. (2022). Optimal estimation of tilt-to-length noise for spaceborne gravitational-wave observatories. J. Guid. Control Dyn..

[26] Hartig M.S., Paczkowski S., Hewitson M., Heinzel G., Wanner G. (2025). Postprocessing subtraction of tilt-to-length noise in LISA in the presence of gravitational wave signals. Phys. Rev. D.

[27] Kenyon S.P., Apple S., Siu J., Wass P.J., Conklin J.W. (2025). Advanced charge control dynamics simulation for the LISA gravitational reference sensor. Class. Quantum Gravity.

[28] Breault Research Organization (2019). ASAP Optical Simulation Software.

[29] MathWorks (2018). MATLAB R2018a.

[30] Morrison E., Meers B.J., Robertson D.I., Ward H. (1994). Experimental demonstration of an automatic alignment system for optical interferometers. Appl. Opt..

